# Exploring salivary cortisol and recurrent pain in mid-adolescents living in two homes

**DOI:** 10.1186/s40359-014-0046-z

**Published:** 2014-10-14

**Authors:** Emma Fransson, Lisa Folkesson, Malin Bergström, Viveca Östberg, Petra Lindfors

**Affiliations:** Centre for Health Equity Studies (CHESS), Stockholm University/Karolinska institutet, 106 90 Stockholm, Sweden; Department of Psychology, Stockholm University, Stockholm, Sweden

**Keywords:** HPA-axis activity, Cortisol, Mid-adolescence, Recurrent pain, Joint physical custody

## Abstract

**Background:**

Each year, around 50.000 children in Sweden experience a separation between their parents. Joint physical custody (JPC), where the child alternates homes between the parents for about equal amount of time, has become a common living arrangement after parental separation. Children in two homes could benefit from everyday contact with both parents and access to both parents’ financial resources. However, children could experience stress from being constantly moving and potentially exposed to parental conflicts. Still, studies on JPC and biological functioning related to stress, are lacking. The aim of this study was to investigate how living arrangements (intact family/JPC) relate to HPA-axis activity and recurrent pain in mid-adolescents.

**Methods:**

Mid-adolescents (106 girls and 51 boys) provided demographic details, self-reports of recurrent pain (headache, stomachache, neck/shoulder and back pain) and salivary samples. Salivary cortisol samples were collected: 1) immediately at awakening, 2) +30 minutes, 3) +60 minutes, and 4) at 8 p.m. The cortisol awakening response (CAR) was computed using an established formula. Additionally, the diurnal decline between the waking and 8 p.m. samples was computed.

**Results:**

Hierarchical multiple regressions showed that living arrangements (intact family/JPC) was not associated with morning cortisol (CAR), the diurnal cortisol decline or with recurrent pain. However, sex was a significant predictor of both cortisol measures and recurrent pain with girls exhibiting a higher cortisol awakening response and a greater diurnal decline value as well as reporting more recurrent pain than did boys.

**Conclusions:**

Living arrangements were not associated with HPA-axis activity or recurrent pain in this group of well-functioning mid-adolescents. Although this study is the first to investigate how living arrangements relate to HPA-axis functioning and additional studies are needed, the tentative findings suggest that these mid-adolescents have adapted to their living arrangements and that other factors play a more pertinent role for HPA-functioning and subjective health.

## Background

Each year, around 50.000 children in Sweden experience a separation between their parents and in 2008, 32 percent of adolescents had divorced parents (Statistics Sweden 2009). Traditionally in Western societies, children have tended to stay with their mothers after parental separation (Kelly 2007; Skjørten et al. 2007). However, the practice of joint physical custody (JPC), where the child alternates homes between the parents for about equal amount of time, is increasing in many Western countries, such as Australia, the U.S and several European countries (Bakker and Mulder 2013; Kaspiew et al. 2011; Lavadera et al. 2013; Melli and Brown 2008; Sodermans et al. 2013). In Sweden about one in ten of all mid-adolescents live in two homes (Bergström et al. 2013), and this practice has been reported as equally common as sole mother custody for recently separated parents (Swedish Government Official Report 2011).

Arguments favoring JPC concern the benefit of keeping close relationships and everyday contact with both parents along with potential benefits of having access to both parents’ financial and social resources (Breivik and Olweus 2006; Skjørten et al. 2007). Concerns involve the potential stress of being constantly moving and perhaps being more exposed to parental conflicts (Bauserman 2002). Additional stress exposures for adolescents in JPC may involve feelings of alienation from living in two separate worlds and daily stressors including long distances to school, friends and leisure activities, instability in parenting and a need to adjust to the demands of two different family lives (Bauserman 2002; Gilmore 2006). Moreover, in an interview study from Australia, children and adolescents in JPC brought up the hassles of leaving things behind in the other home (Cashmore et al. 2010). Despite the increased practice of JPC, research is limited and has primarily focused on adjustment (Bauserman 2002). Studies including health measures have focused on subjective health reports with a recent study (Carlsund et al. 2013) indicating that adolescents living in two homes report more subjective health complaints than do those in intact families. However, studies on JPC and biological functioning are lacking.

Psychosocial stress, in terms of the perception of immediate or long-term challenges or demands, which may be associated with JPC, influences the hypothalamic-pituitary-adrenal (HPA) axis and its regulation of cortisol (O’Connor et al. 2000). Cortisol is important for translating long-term stress into pathological conditions (e.g., Gunnar et al. 2009). Individuals exposed to stress have been found to exhibit for example lower cortisol levels at waking and smaller declines during the day (Fisher et al. 2011) or a greater area under the daytime cortisol curve (Suglia et al. 2010). Specifically, alterations in the diurnal cortisol profiles have been linked to various health problems in children and adolescents (Rotenberg et al. 2012). However, less is known about mid-adolescents’ living arrangements and diurnal cortisol profiles, which motivates exploring further these linkages. Besides influencing physiological functioning, psychosocial stress has also been associated with increasing levels of recurrent pain (Alfvén et al. 2008). Additionally, increased cortisol at awakening has been linked to greater ratings of pain (Goodin et al. 2012). This motivates investigating further the linkages between living arrangements and recurrent pain.

Previous studies have shown differences between girls’ and boys’ reactions following separation. For instance, research has shown that boys may be at increased risk for poorer mental health after parents’ divorce (Malone et al. 2004; Spruijt and Duindam 2005) while girls have been found more negatively affected by losing a father figure (Nielsen 2011) or from being in father custody (Naevdal and Thuen 2004). As for cortisol, differences between adolescent girls and boys have been reported with findings being inconclusive: some studies have shown that stress is associated with larger cortisol expressions in boys (Zijlmans et al. 2013) while other studies have found the same but in girls (de Veld et al. 2012).

In view of the limited research on biological correlates of JPC in adolescents, this study set out to investigate the associations between living arrangements (intact family/JPC) and salivary cortisol in mid-adolescent boys and girls. Drawing on previous research (Carlsund et al. 2013), this study also investigated the associations between living arrangements and subjective health in terms of recurrent pain. Following previous findings, JPC adolescents were hypothesized to report more recurrent pain than their peers in intact families. Due to the regular change of homes and the risk of exposure to parental conflict, we also hypothesized that adolescents in joint physical custody would show different cortisol profiles as compared to those in intact families.

## Methods

### Participants

Adolescents aged 14–16 years were recruited through two compulsory schools in Stockholm, Sweden, to participate in a study of students’ daily life, health and well-being in the 8th and 9th school years. The total population included 545 adolescents. Of these, 413 (246 girls and 167 boys) completed an initial questionnaire that was mandatory for inclusion in the cortisol study. In total, 285 adolescents (166 girls and 119 boys) lived in intact families and 75 (48 girls and 27 boys) in two homes. Relatively few adolescents lived with one parent only and since their contacts with the other parent varied, these adolescents were excluded (*n* = 49). Also, data from adolescents suffering from chronic illness relating to hormonal functioning or taking medication influencing HPA-axis functioning (e.g., asthma and hypothyroidism) were excluded (*n* = 3). Additionally, adolescents (*n* = 15) taking their first sample more than 5 minutes after waking were excluded (Smyth et al. 2013). The present study included 157 adolescents who provided four saliva samples, had complete data on all self-report measures and were living either in intact families or in two homes, moving between their parents (JPC). A comparison of self-rated health and recurrent pain shows no significant differences between the analytic sample and those responding to the questionnaire regarding self-rated health (analytic sample: M = 1.86, SD = .69; others: M = 1.82, SD = .74), or recurrent pain (analytic sample: M =1.96, SD = 2.18; others: M = 1.77, SD = 2.14). Comparisons in self-rated health and recurrent pain between adolescents with different living arrangements who provided saliva samples and those who did not showed no significant group differences.

### Procedure

The research team first implemented the study at the two schools by meeting with school staff and parental representatives. A letter was sent to all of the parents including information about the research project and a form for informed consent regarding participation in a questionnaire study and a biomarker study respectively. Informed consent from a parent was obtained for 83% of the adolescents (parents of 39 adolescents were unavailable). All these adolescents were invited to participate in a questionnaire study. Immediately before completing the questionnaire in the classroom, all adolescents received oral and written information about the study and ten adolescents actively declined participation. The remaining adolescents who were present at school gave written informed consent for the questionnaire. Adolescents returning completed questionnaires were invited to the cortisol study and gave their written informed consent separately for this sub-study. Students returning saliva samples received a voucher (USD 15). About 2/3 of those invited provided at least one saliva sample. This research was approved by the Regional Ethics Committee in Stockholm (Ref. no: 2009/857-31/4).

### Living arrangements and recurrent pain

#### Living arrangements

Regarding demographics, participants were asked to indicate whom they were living with. Those with divorced or separated parents were asked to indicate with yes or no whether they had an additional home and if so how much time they spent in each home. Response alternatives included “half the time”/“less than half the time”/“almost never”. For this study, participants living with both their parents in one home were categorized as intact families. Participants with two homes where they stayed half the time or less than half the time were categorized as having joint physical custody.

#### Recurrent pain

Exposure to stressors is a major risk factor for recurrent pain and co-occurrence of pain, such as headache and abdominal, back and limb pain (see Alfvén et al. 2008). The questionnaire included four items asking about recurrent pain. The question reads as follows: “During the past six months, how often have you had the following problems?”. The items on pain covered headaches, stomachache, back pain and neck/shoulder pain with the response alternatives “every day”, “several times a week”, “once a week”, “sometime during the month” and “less often or never”. Ratings of each item were categorized to show weekly pain/less than weekly pain. A sum score was computed reflecting the number of different pains the participants experienced at least once a week. Thus high scores indicate higher occurrence and co-occurrence of pain (Alfvén et al. 2008).

### Salivary cortisol

This study uses saliva samples collected within two weeks after completing the initial questionnaire, at four points in time during an ordinary school day: 1) immediately at waking up, 2) at 30 minutes post-awakening, 3) at 60 minutes post-awakening, and 4) at 8 p.m. Saliva samples were collected using the Salivette® (Sarstedt Inc., Rommelsdorf, Germany), a plastic tube with a suspended insert containing a sterile neutral cotton wool swab. Adolescents were instructed to chew on the swab for two minutes before putting it back into the tube and sealing it, or to spit saliva directly into the tube, if preferred. Adolescents were also instructed not to eat, smoke, drink coffee/tea (or other beverages containing caffeine), or brush their teeth 10 minutes before sampling saliva (Hanrahan et al. 2006). All samples were stored in plastic-bags in room temperature before returned to the research team on the next school day. Then saliva samples were transported to the laboratory where they were stored in a freezer (-20°C) until analyzed. Cortisol was determined using competitive radioimmunoassay (Spectria Cortisol RIA, Orion Diagnostica, Espoo, Finland; intra-assay precision <5%, 1.7–4.1% and inter-assay precision <10%, 4.3–9.0%). Each sample was analyzed twice and in randomized order with values expressed in nmol/L.

#### Diary data

Each participant was instructed to complete a diary and return it along with the test tubes. Details on time and date for saliva sampling and questions on medication and chronic diseases were included in the diary.

### Statistics

Independent group *t*-tests and ANOVAs were performed to investigate group differences. The main effect of living arrangement (joint physical custody/intact family) was tested using hierarchical blockwise multiple regression analysis. Main effects of demographic characteristics including sex (0 = girl; 1 = boy) and school year (0 = 8th year; 1 = 9th year) were controlled for and entered in the first step. Living arrangements were included in the second step. The third and final step included the interactions between sex∗living arrangements to allow for examination of their effects on each outcome. Due to non-normality, cortisol values were log transformed. The established formula (Pruessner et al. 2003) was then used to compute the cortisol awakening response, CAR with respect to ground. The CAR included the sample collected 1) at waking, 2) 30 min post awakening and 3) 60 min post awakening. Additionally, the diurnal decline was computed using the 1) waking and 4) evening samples. Post hoc power analyses were computed using G*Power 3.1 (Faul et al. 2007).

## Results

Of the mid-adolescents investigated here, 132 (89 girls and 43 boys) were living in intact families and 25 (17 girls and 8 boys) in two homes. Descriptive statistics with means (SD) for cortisol (log transformed values) at different time points for the different groups were as follows; Girls in intact families: at waking: 2.39 (SD = .70); +30 min.: 3.10 (SD = .53); +60 min.: 2.90 (SD = .65); evening: .38 (SD = .82); Girls in two homes: at waking; 2.32 (SD = .72); +30 min.: 2.99 (SD = .44); +60 min.: 2.80 (SD = .47); evening: .50 (SD = 1.00); Boys in intact families: at waking: 1.88 (SD = .71); +30 min.: 2.77 (SD = .57); +60 min.: 2.74 (SD = .46); evening: .31 (SD = .81); Boys in two homes: at waking: 2.24 (SD = .71); +30 min.: 2.79 (SD = .43); +60 min.: 2.74 (SD = .44); evening: .43 (SD = 1.53).

**Figure 1:**
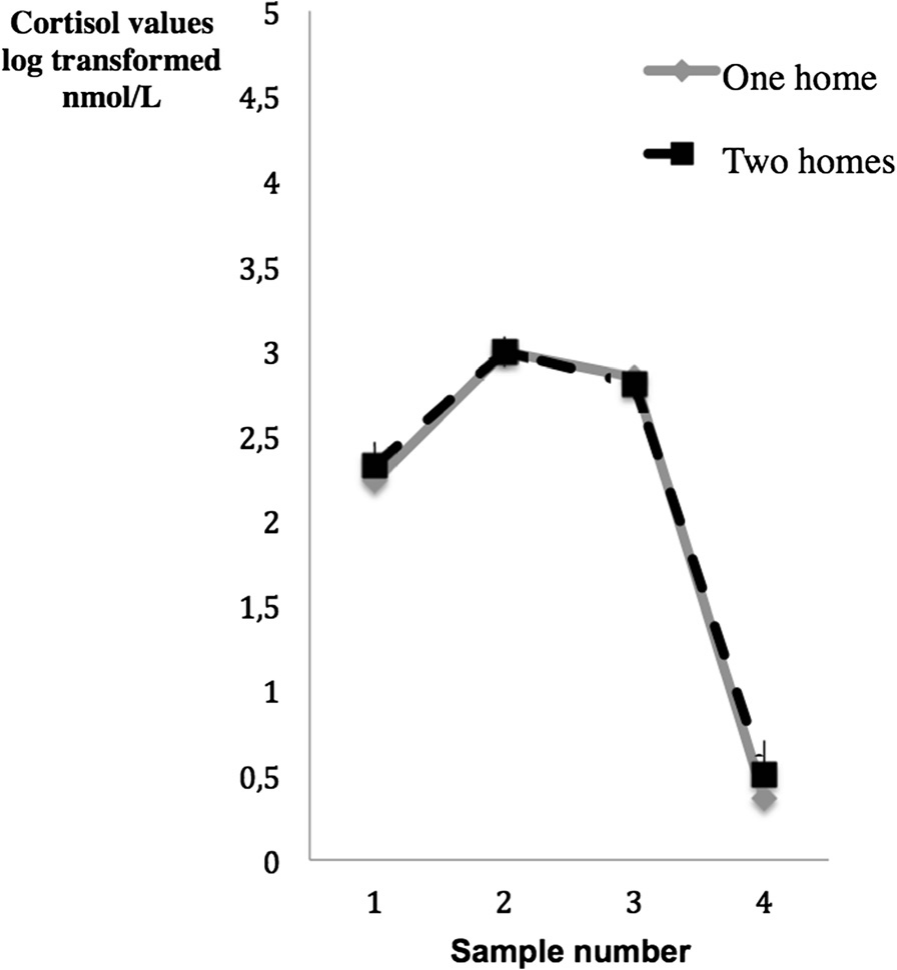
**Means and standard error of means for log transformed cortisol values (nmol/L) at four time points (1 = waking, 2 = +30 min, 3 = +60 min and 4 = at 8 p.m) among adolescents living in one (n = 132) and two homes (n = 25) respectively.**

ANOVAs investigating differences between girls and boys with different living arrangements showed no significant effects of living arrangement for any of the dependent measures. Descriptive statistics for the dependent measures were as follows: The mean CAR for adolescents in intact families was 167.75 (SD = 32.05) while it was 175.74 (SD = 33.03) for those in two homes. For the diurnal decline measure, the mean for adolescents in intact families was 1.87 (SD = 1.10) while it was 1.83 (SD = 1.07) for those in two homes. Figure 1 shows the diurnal variation in cortisol for adolescents with different living arrangements. As for recurrent pain the mean for adolescents in intact families was 1.92 (SD = 2.12) and 1.68 (SD = 2.01) for those in two homes.

Table 1 summarizes results of the hierarchical regressions for the cortisol measures. For cortisol during the first hour of awakening (CAR), sex emerged as a significant predictor across both steps with girls exhibiting a higher cortisol awakening response. Similarly, girls had a greater diurnal decline value. School year, the other predictor entered in the first step, was not significantly associated with any of the cortisol measures. Living arrangements were added in the second step but did not emerge as a significant predictor and there was no increase in the proportion of explained variance. Adding the sex∗living arrangement in the final step did not increase the amount of explained variance and the interaction term was not significantly associated with any of the cortisol measures.

**Table 1:** **Results of hierarchical regression analyses for cortisol measures, unstandardized and standardized regression coefficients (** ***N*** **=157)**

	**CAR**						**Cortisol decline**					
**Predictor**	**Step 1**		**Step 2**		**Step 3**		**Step 1**		**Step 2**		**Step 3**	
	**B**	**Beta**	**B**	**Beta**	**B**	**Beta**	**B**	**Beta**	**B**	**Beta**	**B**	**Beta**
Sex	-24.85***	-.36***	-24.83***	-.36***	-23.73***	-.35***	-.38*	-.17*	-.38*	-.17*	-45*	-.20*
School year	-5.91	-.09	-5.57	-.08	-5.30	-.08	-.07	.03	-.07	-.03	-.09	-.04
Living arrangements			7.39	.08	9.64	.11			-.06	-.02	-.20	-.07
Sex∗living arrangements					-6.95	-.05					.44	.09
R2 adjusted		.13***		.13		.13		.02		.01		.01
R2 change		.14***		.01		.00		.03		.00		.01

*p < .05, ***p < .001.

For CAR, CIs (95%) for the third step were as follows: sex (-34.80/-12.66), school year (-15.13/4.52), living arrangements (-6.23/-25.50), sex∗living arrangements (-34.94/21.04).

For cortisol decline, CIs (95%) for the third step were as follows: sex (-85/-.06), school year (-.44/.26), living arrangements (-77/.37), sex∗living arrangements (-.56/1.45).

Note: Sex: 0 = girls, 1 = boys; School year: grade 8 = 0; grade 9 = 1; Living arrangements: One home = 0, JPC = 1.

As regards recurrent pain (Table 2), sex emerged as a significant predictor with girls reporting more recurrent pain than did boys. School year, however, was a non-significant predictor across both steps. Similar to the cortisol measures, living arrangements did not emerge as a significant predictor and the proportion of explained variance did not increase. Finally, adding the interaction between sex and living arrangement did not increase the amount of explained variance and there was no significant association with recurrent pain.

**Table 2:** **Results of hierarchical regression analyses for recurrent pain, unstandardized and standardized regression coefficients (** ***N*** **=157)**

	**Recurrent pain**					
**Predictor**	**Step 1**		**Step 2**		**Step 3**	
	**B**	**Beta**	**B**	**Beta**	**B**	**Beta**
Sex	-1.23	-.27**	-1.23	-.27**	-1.22	-.27**
School year	.25	.06	.23	.06	.24	.06
Living arrangements			-.23	-.04	-.22	-.04
Sex∗living arrangements					-.02	-.01
R2 adjusted		.07		.06		.08
R2 change		.08		.01		.00

**p < .01.

For recurrent pain, CIs (95%) for the third step were as follows: sex (-1.97/-.47), school year (-.43/.90), living arrangements (-1.30/.85), sex∗living arrangements (-1.92/1.87).

Note: Sex: 0 = girls, 1 = boys; School year: grade 8 = 0; grade 9 = 1; Living arrangements: One home = 0, JPC = 1.

Post hoc power calculations resulted in perceived power as follows: CAR, .02; diurnal decline, .05; recurrent pain, .08.

Taken together, living arrangements did not predict objective or subjective measures. Instead, sex was a significant predictor of the objective cortisol measures and the subjective measure (recurrent pain).

## Discussion

Investigating morning and diurnal HPA-axis activity and recurrent pain, the present findings showed no significant effect of living arrangements on any of these measures. The findings are at odds with the initial hypotheses. However, sex was, in line with previous research, associated with different cortisol profiles over the morning hours and with the diurnal cortisol decline (e.g., Folkesson et al. 2014; Kudielka et al. 2004), with girls exhibiting a higher cortisol awakening response and a greater diurnal decline value. Also, as could be expected, girls reported higher levels of recurrent pain (King et al. 2011; Hjern et al 2008). Yet, to the best of our knowledge, this study is the first study investigating cortisol profiles in adolescents living in two homes, as compared to adolescents in intact families.

Previous studies of linkages between JPC and physical health measures in adolescents (self-reported health complaints and psychosomatic symptoms) suggest a somewhat higher incidence of poor health among JPC adolescents as compared to those in intact families (Carlsund et al. 2013). However, in our sample, consisting of Swedish middle class adolescents, living in two homes might not be a sufficiently strong stressor to induce HPA axis changes or increases in recurrent pain. Yet, recent research shows that children and adolescents in JPC fare better than those living solely with one parent (e.g. Bergström et al. 2013; Bjarnason et al. 2012). In the present study, mid-adolescents living with only one parent were excluded, due to small number of individuals and large variation in contact with the other parent in this group. Compared with other countries, the group living solely with one parent is decreasing. According to a recent Swedish study, 87 per cent of mid-adolescents in non-intact families share their time between two homes even if all of them do not spend equal amounts of time in both homes (Bergström et al. 2013). However, the inconsistency between the present findings on recurrent pain and previous research can perhaps be explained by the fact that the recurrent pain measure used here differs from the subjective health complaint measure used by Carlsund et al. (2013). Specifically, the recurrent pain measure analyzed here is more specific than the measure used in the study by Carlsund and colleagues (2013) which also included mental health complaints.

Other methodological considerations include the cross-sectional design that limits conclusions regarding causality and the lack of details on how long the adolescents have been living in JPC. Still, living arrangements were not related to cortisol. Obviously, the sample size was small which decreases power, hinders separate analyses of girls and boys and increases the risk for false negative findings. Yet, the saliva sampling was monitored through adolescents taking notes of their sampling times and behaviors. This allowed for detailed scrutiny and exclusion of individuals not following sampling instructions. Also, controlling for the time between awakening and the first sample did not influence the findings (results not shown). However, there were no data on menstrual cycle phase or use of oral contraceptives available for the girls meaning that it was impossible to account for the influence of these factors on the cortisol measures. Yet, the differences in cortisol levels between girls and boys found here were in line with previous findings on adolescents (Reynolds et al. 2013). Then again, the cortisol findings may also relate to the fact that mid-adolescents generally are fairly healthy with flexible physiology, which means that the psychosocial stress of living arrangements is yet to be translated into physiological changes influencing HPA-axis functioning and cortisol output.

While previous studies have included more heterogeneous samples, the present study included a homogeneous group living in socio-economically favorable middle-class contexts. Also, this study was conducted in a cultural setting where family policy strives towards parental equality and where welfare policies aim to reduce economical differences between single and cohabiting parents. Obviously, the present findings need replication in larger studies with adequate power that besides JPC adolescents and individuals in intact families also include adolescents living with a single-parent only, a group excluded here because of power reasons. Importantly, future studies on children and adolescents in different family settings need to include measures of biological, psychological and health functioning to describe properly any effects of various living arrangements.

## Conclusions

The present findings showed that living arrangements were not associated with HPA-axis activity or recurrent pain. Although this study is one of the first to investigate how living arrangements relate to HPA-axis functioning and despite the need of additional research, the tentative findings suggest that the mid-adolescents investigated here have adapted to their living arrangements and that other factors, beyond living arrangements, play a more pertinent role for HPA-functioning and subjective health.
